# Estimation of patient flow in hospitals using up-to-date data. Application to bed demand prediction during pandemic waves

**DOI:** 10.1371/journal.pone.0282331

**Published:** 2023-02-27

**Authors:** Daniel Garcia-Vicuña, Ana López-Cheda, María Amalia Jácome, Fermin Mallor

**Affiliations:** 1 Institute of Smart Cities, Public University of Navawordpadrre, Pamplona, Spain; 2 Departamento de Matemáticas, Research Group MODES, CITIC, Universidade da Coruña, A Coruña, Spain; National University of Sciences and Technology NUST, PAKISTAN

## Abstract

Hospital bed demand forecast is a first-order concern for public health action to avoid healthcare systems to be overwhelmed. Predictions are usually performed by estimating patients flow, that is, lengths of stay and branching probabilities. In most approaches in the literature, estimations rely on not updated published information or historical data. This may lead to unreliable estimates and biased forecasts during new or non-stationary situations. In this paper, we introduce a flexible adaptive procedure using only near-real-time information. Such method requires handling censored information from patients still in hospital. This approach allows the efficient estimation of the distributions of lengths of stay and probabilities used to represent the patient pathways. This is very relevant at the first stages of a pandemic, when there is much uncertainty and too few patients have completely observed pathways. Furthermore, the performance of the proposed method is assessed in an extensive simulation study in which the patient flow in a hospital during a pandemic wave is modelled. We further discuss the advantages and limitations of the method, as well as potential extensions.

## 1. Introduction

A key aspect in hospital management is planning strategies to avoid healthcare systems to be overwhelmed, which could involve an increment of the number of preventable deaths. During the COVID-19 pandemic, the explosive growth of the number of infected cases in a short period of time has caused massive strain on medical systems. Although a considerable number of restrictions has been adopted in most countries, hospitals worldwide have been overburdened. Most of the deaths were caused by the virulence of severe acute respiratory syndrome-coronavirus-2 (SARS-CoV-2), but some may have been due to pandemic-associated overloads in hospital capacity [1–4].

The estimation of the hospital ward and Intensive Care Units (ICU) beds’ demand is critical for making wise decisions about clinical operations and resource allocations. Menon et al. [5] expose the importance of estimating the critical care bed capacity, as well as developing an appropriate contingency planning. Specifically, they modelled the demand for critical care beds in England using a range of attack rates and pandemic durations. More recently, Gitto et al. [6] highlight the importance of having a straightforward and data-driven approach which provides accurate predictions of hospital bed demand. In the face of the COVID19 pandemic, a wide variety of recent studies is related to estimating the capacity of hospital and ICU beds around the world. Litton et al. [7] assessed the capacity of ICU beds in Australia, and they report that intensive care bed capacity could be near tripled in response to the expected increase in demand caused by COVID19. Besides, Barasa et al. [8] evaluate the capacity of the Kenyan health system in terms of general hospital and ICU beds. In Europe, Peña and Espinosa [9], Deschepper et al. [10], López-Cheda et al. [11], and Garcia-Vicuña et al. [12], among others, provide different tools for making predictions of the required number of beds in hospital wards and ICUs. This is essential to avoid important ethical dilemmas related to patient triage [13, 14].

Considering all this, it is vital to register not only the updated number of available beds, but also to forecast hospital bed demand. To develop these forecasts, Susceptible, Infected, Recovered (SIR) models [15, 16] or agent-based models (ABMs) [17, 18] have become common tools for estimating demand for hospital beds during the COVID-19 pandemic. The estimation of the number of hospitalized patients is the first step to forecast hospital bed demand in SIR and ABM models. Nonetheless, it is equally important to estimate how the trend of inpatients will be in the near future. This estimation is based on the distribution of the lengths of stay (LoS) of inpatients in hospital ward or ICU, as well as the probabilities of being transferred to the hospital ward or ICU. Discrete Event Simulation (DES) models are being used increasingly in health-care services for the dynamics of the inpatients [19–21]. They assume predetermined parametric models for the distribution of LoS in hospital ward and ICU. DES methods provide reliable and robust estimates, enabling to manage hospital resources in the most efficient way, only if the assumed models conform to the real trajectory of the inpatients in the hospital facilities. Consequently, it is crucial to obtain accurate estimations used in the simulation models to obtain solid forecasts which would support healthcare managers in optimal resource planning, especially in times of pandemics when healthcare resources are scarce.

In the literature, model parameters for the estimation of the lengths of stay are usually derived from published data [15] and using the health system’s historical data [22]. This results in a non-dynamic static model. Nonetheless, the course of a pandemic is a non-stationary situation, in which hospitalization parameters may vary between different waves and places, and evolve over time. Integration of near-real-time hospital occupancy data into the model can have a large impact on improving forecast accuracy [23].

**Fig 1:**
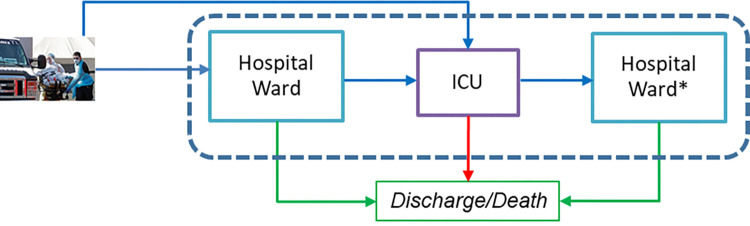
Representation of patient flow in the health system. The symbol * represents that those patients have been in the ICU before.

Hospital electronic health record systems provide patient-level information that allows knowing both the pathway of each released patient, and their current location (ward or ICU) if they have not been discharged. Each patient arriving at the hospital can be admitted to the hospital ward or directly to the ICU. Besides, those patients admitted to the wards may worsen their health status and require the transfer to the ICU. From both facilities, patients can die, so they abandon the system, or they can be discharged after improving their health status. In the last situation, patients in the ICU would be transferred to the hospital ward (we use the symbol * to represent that those patients have been in the ICU before) until they get over the disease (see Fig 1).

In this paper, we consider the problem of estimating the distribution of variables associated with the pathway and LoS of patients in hospital ward and ICU dynamically. In contrast to standard analysis where data are analyzed after the end-of-study, in this application, the end-of-study is a moving target. We propose to estimate them along the time by using all available data collected during a moving time window, from the beginning of the pandemic the first infected patient was admitted to hospital to day *t* after the pandemic started.

The major challenge of the proposed methodology is how to handle the information from patients still hospitalized, since only the pathway of the discharged patients is fully known. This lack of complete information of the inpatients is due to not only censorship in the observed LoS but also to the fact that it is unknown which event will be observed in the future. The main contribution of this paper is the introduction of some new approaches which deal with this challenge.

The objectives in this work are twofold: (a) to propose two competitive methods to estimate the probability distributions included in the patient pathway, which take advantage of the incomplete information from patients still in hospital at the time of the estimation; (b) to compare these methods with alternatives that dismiss the valuable information of these inpatients. The performance of the proposed and alternative estimators is assessed in a simulation study. Furthermore, using the ICU bed prediction method in [12], the efficiency of these predictions on the accuracy of the statistical estimators is evaluated.

The rest of the paper is organized as follows. Section 2 introduces the new methodology and describes the notation. Section 3 presents the design of experiments for the simulations. The results obtained in the two simulation studies are included in Section 4. Finally, Section 5 ends the paper with the conclusions of this work.

## 2. Methodology

### 2.1. The estimation problem

**Fig 2:**
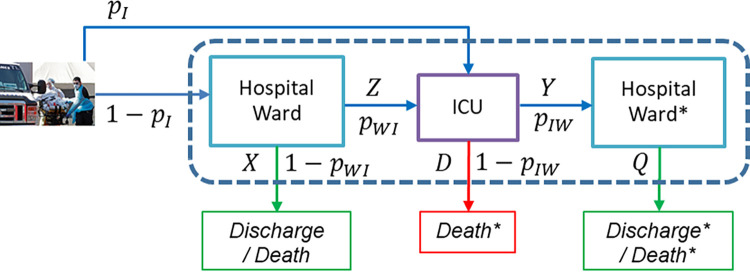
Representation of patient flow in the health system showing the LoS variables and branching probabilities. *Z*: time in hospital ward until admission to the ICU, *X*: time in hospital ward until discharge or death, *Y*: time in the ICU before being transferred to hospital ward, *D*: time in the ICU until death, and *Q*: time in the hospital ward after discharge from the ICU and the branching probabilities *p*_*I*_: probability of direct admission to the ICU, *p*_*WI*_: probability of admission to the ICU from the ward, and *p*_*IW*_: probability of going to hospital ward from ICU. The symbol * represents that those patients have been in the ICU before.

We consider the problem of forecasting hospital bed demand by estimating the distribution of the LoS and probabilities associated with the pathway of patients hospitalized during a pandemic wave. Because LoS probability distributions and branching probabilities may vary between different waves and between different places, we propose a method to estimate them that uses all data collected from the time the first infected patient was admitted, until the present time. Patients who have already abandoned the hospital due to discharge or death provide complete information for the estimation of LoS probability distributions and branching probabilities, while patients who are still hospitalized provide censored information that may not even be known to which variables are referred to, as we explain below. At the beginning of a pandemic wave, there are few patients and most of them are still hospitalized, but their valuable information should not be disregarded by the statistical estimators. Fig 2 shows the same patient flow as Fig 1, including the LoS probability distributions and the branching probabilities that compose the patient pathway through the hospital.

**Fig 3:**
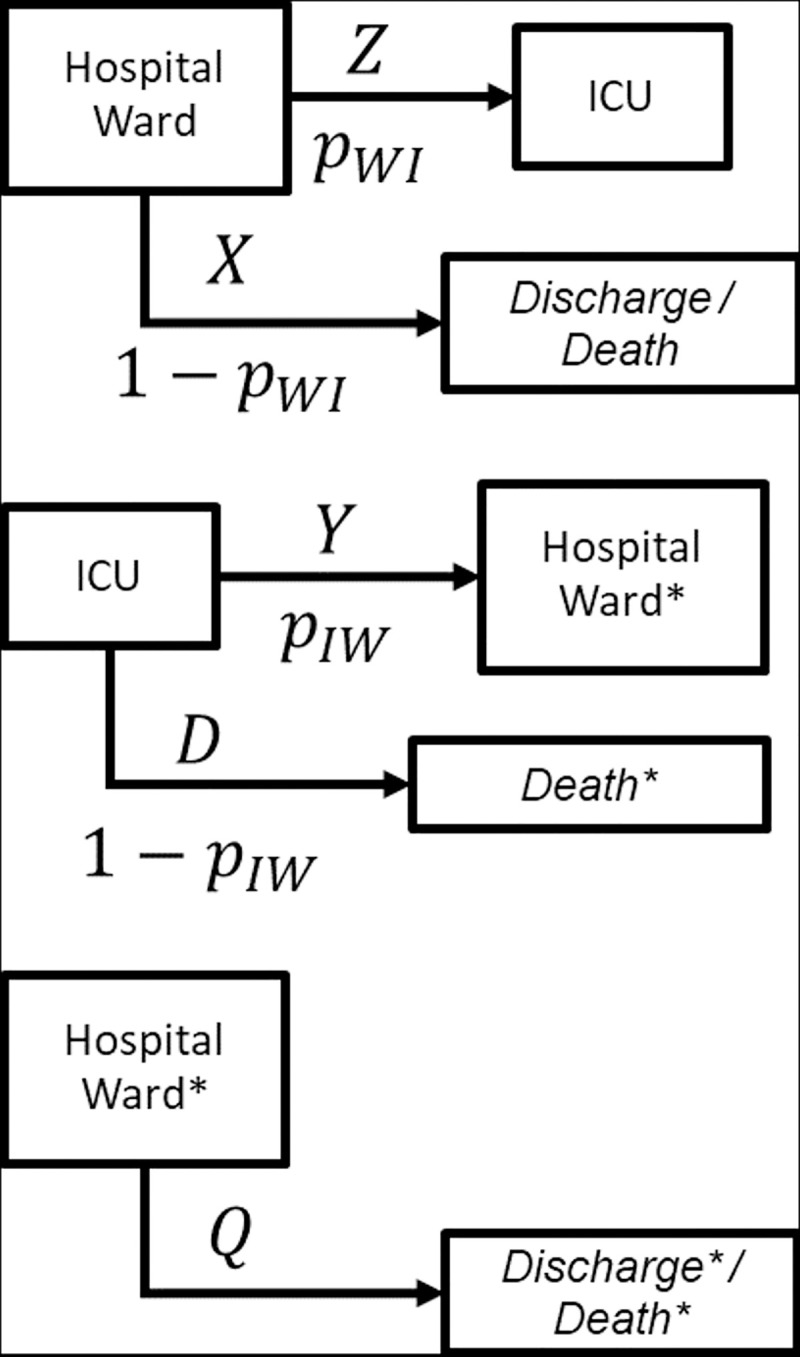
Flow diagram of patients in three situations. Patients admitted to the hospital (top), patients admitted to the ICU (center), and patients discharged from the ICU (bottom).

For a patient who has been hospitalized in hospital ward, it is unknown whether he or she will be finally admitted to the ICU or not, so it is unknown whether the observed value of the LoS in hospital ward is a censored observation for the variable *Z*, “time in hospital ward until admission to the ICU”, or for the variable *X*, “time in hospital ward until discharge or death without ICU admission”. In this section, we propose an estimation method for the probability distributions of these variables *Z* and *X*, as well as the probability of admission to the ICU from the ward, *p*_*WI*_, that uses the information of all patients admitted to the hospital at the present time (Fig 3, top).

Observe that the same estimation methodology can be applied to the estimation of the probability distributions of *Y*, “time in the ICU before being transferred to hospital ward”, and *D*, “time in the ICU until death”, and *p*_*IW*_, the probability of discharge to hospital ward (Fig 3, center). In this case, it is unknown whether a patient who is admitted to the ICU will evolve favourably until being transferred to the hospital ward or whether he or she will die in the ICU. Therefore, the observed LoS of these patients still in the ICU is censored, and it is unknown if it is a censored observation of *Y* or *D*. Finally, patients discharged from the ICU and still admitted to the hospital ward provide censored data for the variable *Q* “time in the hospital ward after being discharged from the ICU” (Fig 3, bottom). The estimation of the variable *Q* can be obtained with traditional methods that deal with censoring.

From this point onwards, the introduced notation and methods correspond to the estimation of the distribution of variables *Z* and *X* and probability *p*_*WI*_ (Fig 3, top) and refer to patients admitted to hospital ward, so patients admitted directly to ICU are not considered. Same methods with similar notation must be used with patients admitted to ICU for the estimation of the distribution of variables *Y* and *D* and probability *p*_*IW*_ (Fig 3, center). The estimation of the distribution of variables *Q* (Fig 3, bottom) can be performed with classical methods in survival analysis.

Time *t* = 0 is set as the time the first patient is admitted to the hospital. At a fixed time *t*, each of the *n*(*t*) patients can be in one of the following sets: (a) ICU set: patients who have required ICU at some point, regardless if they are still in ICU, returned to hospital ward (Hospital Ward* in Fig 2) or discharged (Discharge*/Death* in Fig 2); (b) HW set: patients without ICU admission who still are in the hospital ward (Hospital Ward in Fig 2); and (c) DIS set: patients without ICU admission already discharged (Discharge/Death in Fig 2).

For each patient *i*, with *i* = 1,…,*n*(*t*), admitted to the hospital before time *t*, we define a vector $u_{i}(t)=[{t_{HA}}_{i},{t_{HD}}_{i},{t_{IA}}_{i},{t_{ID}}_{i}]$ that contains four times: ${t_{HA}}_{i}$ the time of admission to hospital ward, ${t_{HD}}_{i}$ the time of discharge from hospital ward, ${t_{IA}}_{i}$ the time of admission to ICU, and ${t_{ID}}_{i}$ the time of discharge from ICU. At a fixed time *t*, the patient *i* can be still in the hospital ward or in the ICU, so in these cases ${t_{HD}}_{i}=t$ and ${t_{ID}}_{i}=t$ respectively. Besides this, some of the times in ***u***_***i***_(*t*) might remain unknown. For example, if the patient *i* was not admitted to ICU at time *t*, then ${t_{IA}}_{i}\;\mathrm{and}\;{t_{ID}}_{i}$ are unknown and they will be denoted as ∅.

The times in vector ***u***_***i***_(*t*) enable the patients to be classified into the aforementioned sets: (a) ICU set: patients with ICU admission divided into three subsets, those back to the hospital ward from ICU with $u_{i}(t)=[{t_{HA}}_{i},t,{t_{IA}}_{i},{t_{ID}}_{i}]$, those still in ICU with $u_{i}(t)=[{t_{HA}}_{i},t,{t_{IA}}_{i},t]$, and with $u_{i}(t)=[{t_{HA}}_{i},{t_{HD}}_{i},{t_{IA}}_{i},{t_{ID}}_{i}]$ those who have died in the ICU (${t_{HD}}_{i}={t_{ID}}_{i}$) and those who have already been discharge from hospital ward (${t_{HD}}_{i}>{t_{ID}}_{i}$); (b) HW set: patients in hospital ward without ICU admission with admission dates $u_{i}(t)=[{t_{HA}}_{i},t,\emptyset ,\emptyset ]$; and (c) DIS set: discharged patients who did not required ICU with $u_{i}(t)=[{t_{HA}}_{i},{t_{HD}}_{i},\emptyset ,\emptyset ]$.

Let us define the indicator of the event `admission to ICU´, given by *δ*_*i*_(*t*) = 1 if ${t_{IA}}_{i}$ is known at time *t* (ICU set) and *δ*_*i*_(*t*) = 0 otherwise (HW and DIS sets). Similarly, let us denote *ν*_*i*_(*t*) the indicator which reveals if the patient has been discharged directly from hospital ward or died at a time before *t*, so ICU admission will never be required. In other words, *ν*_*i*_(*t*) = 1 if the patient belongs to DIS set ($\left({t_{IA}}_{i},{t_{ID}}_{i})=(\emptyset ,\emptyset \right)$ and ${t_{HD}}_{i}$ is known), and *ν*_*i*_(*t*) = 0 otherwise (HW and ICU sets). We consider the trivariate variable *O* = (*T*,*δ*,*ν*), where *T*, a variable related to the observed length of stay in hospital ward, may take the following values:

$$\begin{array}{l} t_{i}=t_{HD_{i}}-t_{HA_{i}}\;\mathrm{when}\;\delta_{i}(t)=0\;\mathrm{and}\;v_{i}(t)=1\;(\mathrm{DIS}\;\mathrm{set})\\ t_{i}=t_{IA_{i}}-t_{HA_{i}}\;\mathrm{when}\;\delta_{i}(t)=1\;\mathrm{and}\;v_{i}(t)=0\;(ICU\;\mathrm{set})\\ t_{i}=t-t_{HA_{i}}\;\mathrm{when}\delta_{i}(t)=0\;\mathrm{and}v_{i}(t)=0\;(\mathrm{HW}\;\mathrm{set}) \end{array}$$ (1)

Observe that, at a time *t*, value *t*_*i*_ of patients in DIS set provides an observation of variable *X*, value *t*_*i*_ of patients in ICU set provides an observation of variable *Z*, and value *t*_*i*_ of patients in HW set provides a censored observation for either variable *X* or variable *Z*.

For the rest of the paper, we consider the following notation:

- ${\hat{p}}_{WI}(t)$ the estimation of the probability *p*_*WI*_ at time *t*.
- *F*_*X*_(*x*), *F*_*Z*_(*z*) the cumulative distribution function of variables *X* and *Z*, respectively.

### 2.2. Nonparametric methods using survival analysis

Survival analysis refers to the statistical methods used to analyze time-to-event data in the presence of censored observations. Note that the information related to patients in states ICU and DIS is complete for the estimation of the distributions of *Z* and *X*, respectively. However, for patients still in HW set at time *t*, we have right censored data since it is unknown whether they will require ICU or not, nor the final duration of the stay in hospital ward. It should be noted that, in the first weeks of the pandemic, HW set is expected to include most patients. All patients in hospital ward at time *t* provide valuable information for the estimation of *p*_*WI*_ and the distribution of *X* and *Z*. It is therefore essential to carry out a methodology which incorporates all the information contained in these censored observations.

#### NP method

Nonparametric (NP) methods for estimation have specific advantages such as flexibility and ease of computation, and are a popular choice for analysing survival data, such as the Kaplan-Meier estimator to estimate the survival function or the Nelson-Aalen estimator for the cumulative hazard function.

In classical survival analysis, it is assumed that all the individuals will experience the event of interest. That is the case when estimating the distribution of the variable *Q* (Fig 3, bottom) as the event of interest is `Discharge*/Death*´, and this model assumes that all patients in hospital ward coming from ICU will never require ICU again and leave hospital eventually. Therefore, classical nonparametric methods in survival analysis, such as Kaplan-Meier estimator, can be used to estimate the distribution of *Q*. However, that assumption does not hold for the estimation of the distribution of *Z*, “time in hospital ward until admission to the ICU”, since the event of interest is `ICU admission´ and not all the inpatients will require entering ICU. The same situation holds when estimating the distribution of *X*, “time in hospital ward until discharge without ICU admission”, as not all the patients in hospital ward will be discharged without ICU admission. The individuals who are free of experiencing the event are called *long-term survivors*, or simply *cured subjects*. Note that here a *cured* individual refers to a subject who will not experience the event of interest, and this is not necessarily related to be *cured* in medical terms.

Mixture cure models (MCM) account for this situation since they consider that the population is a mixture of two groups of patients, the susceptible ones to the event of interest and the cured individuals (see [24–28] among others). The observed time of all cured individuals is censored, as the event will not occur and therefore it is never observed. Traditional MCM assume that cured individuals are unidentifiable, as censoring prevents from distinguishing which censored subjects are cured and which ones will experience the event in the end. Nonetheless, that is not the case in our context. MCM when the cured subjects are randomly identified addresses this situation, and it has received much attention in recent years (see [29–32]).

When estimating the distribution of *Z*, “time in hospital ward until admission to the ICU”, all patients admitted to ICU (*δ*_*i*_(*t*) = 1) are uncensored while those who have already been discharged from hospital at a time before *t* without ICU admission (*ν*_*i*_(*t*) = 1) are cured from the event `ICU admission´ as they will never be admitted to ICU in the future. For a fixed time *t*, the *NP method* estimates the distribution function of *Z*, *F*_*Z*_(*z*) = *p*(*Z*≤*z*), nonparametrically using the estimator in Safari et al. [33] and the observations $\left\{\left(t_{i},\delta_{i}(t),\nu_{i}(t)),i=1,\ldots ,n(t\right)\right\}$

$${\hat{F}}_{Z,t}^{NP}(z)=1-\frac{{\tilde{F}}_{Z,t}(t_{n(t)})-{\tilde{F}}_{Z,t}(z)}{{\tilde{F}}_{Z,t}(t_{n(t)})},$$

$$\mathrm{where}\;{\tilde{F}}_{Z,t}(z)=1-\prod_{i=1}^{n(t)}\left\{1-\frac{\delta_{i}(t)1(t_{i}\leq z)}{n(t)-i+1+\sum_{j=1}^{i-1}\nu_{j}(t)}\right\}$$

where *t*_1_≤⋯≤*t*_*n*(*t*)_ are the sorted observed times in Eq (1).

Similarly, when estimating the distribution of *X*, “time in hospital ward until discharge without ICU admission”, all patients discharged or dead without ICU admission (*ν*_*i*_(*t*) = 1) are uncensored, and those admitted to ICU (*δ*_*i*_(*t*) = 1) are cured from the event because they will never experience `discharge without ICU admission´. The distribution function of the time in hospital ward until discharge without ICU admission, *F*_*X*_(*x*) = *P*(*X*≤*x*) is estimated nonparametrically for a fixed time *t*, as follows;

$${\hat{F}}_{X,t}^{NP}(x)=1-\frac{{\tilde{F}}_{X,t}(t_{n(t)})-{\tilde{F}}_{X,t}(x)}{{\tilde{F}}_{X,t}(t_{n(t)})},$$

$$\mathrm{where}\;{\tilde{F}}_{X,t}(x)=1-\prod_{i=1}^{n(t)}\left\{1-\frac{\nu_{i}(t)1(t_{i}\leq x)}{n(t)-i+1+\sum_{j=1}^{i-1}\delta_{j}(t)}\right\}$$

Finally, the probability of requiring ICU from ward is estimated for a fixed time *t* using the nonparametric estimator in Safari [32]:

$${\hat{p}}_{WI}^{NP}(t)=1-\prod_{i=1}^{n(t)}\left\{1-\frac{\delta_{i}(t)}{n(t)-i+1+\sum_{j=1}^{i-1}\nu_{j}(t)}\right\}.$$ (2)

See [32, 33] for the consistency and order of convergence of estimators (1) and (2).

There are some nonparametric alternatives to estimate the probability *p*_*WI*_, such as imputation methods [34] or a competing risks model [29]. Note that the first method is biased under the common assumption of independent censoring. Besides, the main disadvantage of the second approach is that, if the patient with the largest observed time is still in hospital ward and did not require ICU (HW set), then the estimator of *p*_*WI*_ is not unique, and only upper and lower bounds are provided [32].

Standard and routinely-implemented cure model methodologies, such as the mixture cure model based on the proportional hazards assumption [35–39] or the accelerated failure time model [40–44] are not discussed here since covariates are not considered in the model.

### 2.3. Parametric methods based on the EM algorithm

We denote as ***o***(*t*) = (*o*_1_(*t*),… *o*_*i*_(*t*),… *o*_*n*(*t*)_(*t*)) the realization of variable *O* in the *n*(*t*) patients admitted to the hospital since the beginning of the pandemic wave. We have developed an iterative procedure, based on the Expectation-Maximization (EM) algorithm, to estimate the distribution functions of the variables *X* and *Z* and the probability *p*_*WI*_. First, an initial estimation of the parameters is carried out by using only the fully-known data, those observations with *δ*_*i*_(*t*)+*ν*_*i*_(*t*) = 1, that is, in DIS (*ν*_*i*_(*t*) = 1) or ICU (*δ*_*i*_(*t*) = 1) sets. In the main iteration, the estimated parameters are used to update the probability of being admitted to ICU for each patient in HW set. These updated probabilities allow the calculation of a new likelihood function for the parameters, which is maximized to obtain a new estimation of the probability distribution parameters. These two steps (updating ICU admission probabilities and getting and maximizing the new likelihood function) are repeated until the stopping criteria are satisfied.

We consider the following additional notation:

- *θ*_*V*_ the vector of parameters of the distribution function of a general variable *V*.
- ${\hat{\theta }}_{V}(t)$ the estimation of the vector of parameters *θ*_*V*_ at time *t*.
- $F_{\theta_{V}}(v),\;f_{\theta_{V}}(v)$ the distribution and density function of a general variable *V* with parameters *θ*_*V*_ respectively.
- *L*_*V*_(*θ*_*V*_|***o***(*t*)) the likelihood function of sample ***o***(*t*) used to estimate *θ*_*V*_.
- ${\hat{\theta }}_{X}^{\left(k\right)}(t)$ and ${\hat{\theta }}_{Z}^{\left(k\right)}(t)$: the estimation of vectors *θ*_*X*_ and *θ*_*Z*_ in the *k*-th iteration of the algorithm at time *t*.
- ${\hat{p}}_{WI}^{\left(k\right)}(t)$: the estimation of the probability *p*_*WI*_ in the *k*-th iteration of the algorithm at time *t*.

The steps of the algorithm are detailed below in the *EM method*.

#### EM method

*1*. *Initialization*. We set *k* = 0 and estimate the parameters *θ*_*X*_, *θ*_*Z*_ and the probability *p*_*WI*_ by using the data in vector ***o***(*t*):

$${\hat{p}}_{WI}^{\left(0\right)}(t)=\frac{\sum_{i=1}^{n(t)}\delta_{i}(t)}{\sum_{i=1}^{n(t)}\left(\delta_{i}(t)+\nu_{i}(t)\right)},$$ (3)

$${\hat{\theta }}_{X}^{\left(0\right)}(t)=\arg\;\underset{\theta_{X}}{\max}\;L_{X}^{\left(0\right)}(\theta_{X}|o(t)),\;where\;L_{X}^{\left(0\right)}(\theta_{X}|o(t))=\prod_{i=1}^{n(t)}{f_{\theta_{X}}(t_{i})}^{\nu_{i}(t)},$$ (4)

$${\hat{\theta }}_{Z}^{\left(0\right)}(t)=\arg\;\underset{\theta_{Z}}{\max}\;L_{Z}^{\left(0\right)}(\theta_{Z}|o(t)),\;where\;L_{Z}^{\left(0\right)}(\theta_{Z}|o(t))=\prod_{i=1}^{n(t)}{f_{\theta_{Z}}(t_{i})}^{\delta_{i}(t)}.$$ (5)

*2*. *Repeat until stop criteria are met*. Iteration *k*+1. From the *k*-th iteration, *k*≥0, the estimations ${\hat{\theta }}_{X}^{\left(k\right)}(t)$, ${\hat{\theta }}_{Z}^{\left(k\right)}(t)$ and ${\hat{p}}_{WI}^{\left(k\right)}(t)$ are known. The iteration is divided in two steps: in the first one, the calculation of the expected value of the probability of admission to the ICU of each patient in HW set is carried out, which allows estimating the probability of admission to ICU, *p*_*WI*_, and the expectation of the likelihood function. The second step computes the estimations of *θ*_*X*_ and *θ*_*Z*_ by maximizing the likelihood functions in the previous step.

***2*.*1*. *Expectation*.** For each patient *i* in HW set, the probability ${\hat{p}}_{WI,i}^{\left(k+1\right)}(t)$ of being admitted to ICU is updated as the posterior probability given the time *t*_*i*_ already spent at the hospital ward:

$${\hat{p}}_{WI,i}^{\left(k+1\right)}(t)\equiv P\left(\delta_{i}(s)=1,s>t|{\hat{p}}_{WI}^{\left(k\right)}(t),{\hat{\theta }}_{X}^{\left(k\right)}(t),{\hat{\theta }}_{Z}^{\left(k\right)}(t)\right)=\frac{\left(1-F_{{\hat{\theta }}_{Z}^{\left(k\right)}(t)}(t_{i})){\hat{p}}_{WI}^{\left(k\right)}(t\right)}{\left(1-F_{{\hat{\theta }}_{Z}^{\left(k\right)}(t)}(t_{i})){\hat{p}}_{WI}^{\left(k\right)}(t)+(1-F_{{\hat{\theta }}_{X}^{\left(k\right)}(t)}(t_{i}))(1-{\hat{p}}_{WI}^{\left(k\right)}(t)\right)}.$$

Considering the updated probabilities of being admitted to ICU for each patient in HW set, we estimate the unconditional probability of admission to ICU:

$${\hat{p}}_{WI}^{\left(k+1\right)}(t)=\frac{1}{n(t)}\sum_{i=1}^{n(t)}\left[\delta_{i}(t)+(1-\delta_{i}(t))(1-\nu_{i}(t)){\hat{p}}_{WI,i}^{\left(k+1\right)}(t)\right]$$

and the likelihood functions of the sample as expected functions:

$$L_{X}^{\left(k+1\right)}(\theta_{X}|o(t))=E[L_{X}(\theta_{X}|o(t))]=\prod_{i=1}^{n(t)}{f_{\theta_{X}}(t_{i})}^{\nu_{i}(t)}\prod_{i=1}^{n(t)}\left[{\left(1-F_{\theta_{X}}(t_{i})\right)}^{\left(1-\delta_{i}(t))(1-\nu_{i}(t)\right)}(1-{\hat{p}}_{WI,i}^{\left(k+1\right)}(t))\right],$$

$$L_{Z}^{\left(k+1\right)}(\theta_{Z}|o(t))=E[L_{Z}(\theta_{Z}|o(t))]=\prod_{i=1}^{n(t)}{f_{\theta_{Z}}(t_{i})}^{\delta_{i}(t)}\prod_{i=1}^{n(t)}\left[{\left(1-F_{\theta_{Z}}(t_{i})\right)}^{\left(1-\delta_{i}(t))(1-\nu_{i}(t)\right)}{\hat{p}}_{WI,i}^{\left(k+1\right)}(t)\right].$$

***2.2*. *Maximization*.** The likelihood functions are maximized to find the parameter estimation:

$${\hat{\theta }}_{X}^{\left(k+1\right)}(t)=arg\;\underset{\theta_{X}}{\max}(L_{X}^{\left(k+1\right)}(\theta_{X}|o(t))),$$

$${\hat{\theta }}_{Z}^{\left(k+1\right)}(t)=arg\;\underset{\theta_{Z}}{\max}(L_{Z}^{\left(k+1\right)}(\theta_{Z}|o(t))).$$

*3*. *Stop criteria*. Let *ε*_*X*_, *ε*_*Z*_, and $\varepsilon_{p_{WI}}$ be some fixed values that control the accuracy of the iterative calculations. Repeat Step 2 until the sequence of values of the estimated parameters converges:

$$|{\hat{\theta }}_{X}^{\left(k+1\right)}(t)-{\hat{\theta }}_{X}^{\left(k\right)}(t)|\leq \varepsilon_{X},$$

$$|{\hat{\theta }}_{Z}^{\left(k+1\right)}(t)-{\hat{\theta }}_{Z}^{\left(k\right)}(t)|\leq \varepsilon_{Z},$$

$$|{\hat{p}}_{WI}^{\left(k+1\right)}(t)-{\hat{p}}_{WI}^{\left(k\right)}(t)|\leq \varepsilon_{p_{WI}}.$$

The final estimates are ${\hat{p}}_{WI}^{EM}(t)={\hat{p}}_{WI}^{\left(k+1\right)}(t),\;{\hat{\theta }}_{X}^{EM}(t)={\hat{\theta }}_{X}^{\left(k+1\right)}(t)$, and ${\hat{\theta }}_{Z}^{EM}(t)={\hat{\theta }}_{Z}^{\left(k+1\right)}(t)$, and then ${\hat{F}}_{X,t}^{EM}(x)=F_{{\hat{\theta }}_{X}^{EM}(t)}(x)$ and ${\hat{F}}_{Z,t}^{EM}(z)=F_{{\hat{\theta }}_{Z}^{EM}(t)}(z)$.

#### EMNP method

Different estimators considered for the initialization step (*k* = 0) result in a different method for the final estimators of the parameters *θ*_*X*_ and *θ*_*Z*_ and the probability of admission to ICU from ward, *p*_*WI*_. The *EMNP* method combines both the *EM* algorithm and the nonparametric approach. This integrated approach is intended to consider the flexibility of the *NP* method and the efficiency of the *EM* algorithm. Specifically, the probability *p*_*WI*_ is initially estimated using the NP estimator in Eq (2), that is, ${{\hat{p}}_{WI}^{\left(0\right)}(t)=\hat{p}}_{WI}^{NP}(t)$. The initial values for the parameters *θ*_*X*_ and *θ*_*Z*_ are those in Eqs (4)–(5). The other steps in the EM method remain unchanged. Finally, the EMNP estimators are ${\hat{p}}_{WI}^{EMNP}(t),\;{\hat{\theta }}_{X}^{EMNP}(t)$, and ${\hat{\theta }}_{Z}^{EMNP}(t)$, given by the EM algorithm, and then ${\hat{F}}_{X,t}^{EMNP}(x)=F_{{\hat{\theta }}_{X}^{EMNP}(t)}(x)$ and ${\hat{F}}_{Z,t}^{EMNP}(z)=F_{{\hat{\theta }}_{Z}^{EMNP}(t)}(z)$.

### 2.4. Naïve alternative methods

We present three naïve alternative methods for estimating the distribution parameters *θ*_*X*_ and *θ*_*Z*_ and the probability *p*_*WI*_. They do not consider censored observations, that is, patients in HW set by time *t*. Although it results, at the beginning of the pandemic wave, in possibly biased estimates, this negative effect tends to fade away at advanced stages of the pandemic, as the number of censored observations decreases. The first method only uses complete information (CI *method*), that is, it only includes those patients admitted to the hospital whose values of the vector ***u***_*i*_(*t*) are completely known. In an attempt to increase the sample size, we define two estimation procedures that somehow include the censored observations given by patients still in hospital ward who have not required ICU yet (HW set). On the one hand, by assuming that all these patients in HW set will not require ICU in the future (I *method*). This method is expected to be biased as long as the assumption is not true. The last estimation procedure (IP *method*) reduces estimation bias by considering all the patients with complete information and some of the patients currently admitted in the hospital ward with unknown entrance to the ICU.

#### CI method

Only patients who entered ICU or have been discharged are included in the estimations, so patients in HW set by time *t* are dismissed. This results in ${\hat{p}}_{WI}^{CI}(t)={\hat{p}}_{WI}^{\left(0\right)}(t),\;{\hat{\theta }}_{X}^{CI}(t)={\hat{\theta }}_{X}^{\left(0\right)}(t)$, and ${\hat{\theta }}_{Z}^{CI}(t)={\hat{\theta }}_{Z}^{\left(0\right)}(t),$ the initial estimations for the EM method in Eqs (3)–(5). So ${\hat{F}}_{X,t}^{CI}(x)=F_{{\hat{\theta }}_{X}^{CI}(t)}(x)$ and ${\hat{F}}_{Z,t}^{CI}(z)=F_{{\hat{\theta }}_{Z}^{CI}(t)}(z)$.

This approach of omitting the observations in HW set raises several issues. First, it leads to the loss of valuable information. Second, the result of ignoring these censored observations is an underestimation of the distributions of *Z* and *X*, since only the patients who have been quickly discharged or transferred to ICU will be considered in the procedure. This underestimation, of considerable magnitude at early stages of the pandemic given the large number of censored observations in the data, will ease over time as the proportion of censored observations decreases.

#### I method

The estimation of parameter *θ*_*Z*_ for the distribution of *Z*, the length of stay in hospital ward until ICU admission, is the same as in the CI method, ${\hat{\theta }}_{Z}^{I}(t)={\hat{\theta }}_{Z}^{\left(0\right)}(t)$ in Eq (5) (${\hat{F}}_{Z,t}^{I}(z)=F_{{\hat{\theta }}_{Z}^{I}(t)}(z)$). As for the estimation of *θ*_*X*_ and the probability *p*_*WI*_, this method seeks to include the censored information of the patients in HW set. The final event of these inpatients remains unknown by time *t*, but most patients still in HW set are expected not to require ICU in the future. This method oversimplifies the model by assuming that none of these patients in HW set will be admitted to the ICU. Therefore, the probability *p*_*WI*_ is estimated empirically at time *t* as follows:

$${\hat{p}}_{WI}^{I}(t)=\frac{\sum_{i=1}^{n(t)}\delta_{i}(t)}{n(t)}$$ (6)

Regarding the estimation of parameter *θ*_*X*_ for the distribution of the length of stay in hospital ward until discharge, *X*, all the observed LoS of the patients in HW set ((*δ*_*i*_(*t*), *ν*_*i*_(*t*)) = (0,0)) by time *t*, $t_{i}=t-{t_{HA}}_{i},$ are considered as censored observations of variable *X*:

$${\hat{\theta }}_{X}^{I}(t)=\arg\;\underset{\theta_{X}}{\max}\;L_{X}^{I}(\theta_{X}|o(t)),\;where\;L_{X}^{I}(\theta_{X}|o(t))=\prod_{i=1}^{n(t)}{f_{\theta_{X}}(t_{i})}^{\nu_{i}(t)}\prod_{i=1}^{n(t)}{\left(1-F_{\theta_{X}}(t_{i})\right)}^{\left(1-\delta_{i}(t))(1-\nu_{i}(t)\right)}$$ (7)

Therefore, ${\hat{F}}_{X,t}^{I}(x)=F_{{\hat{\theta }}_{X}^{I}(t)}(x)$. Note that these I estimators are biased. In fact, both the probability of being transferred to the ICU from ward (*p*_*WI*_) and the time until transfer to the ICU (*Z*) are underestimated. Observe that some patients in HW set will require admission to the ICU so their observed LoS *t*_*i*_, used in Eq (7) as censored observations of *X*, are actually censored values for variable *Z*. This yields biased estimates of the parameters *θ*_*X*_ and *θ*_*Z*_. In turn, ${\hat{p}}_{WI}^{I}(t)$ underestimates the probability of admission to ICU from ward as only patients in ICU set are included in the numerator of Eq (6), while some patients in the HW set will be admitted to ICU as well. Nonetheless, the estimations will improve as pandemic advances, and ICU and DIS sets grow in size with respect to HW set.

**IP method.** In order to reduce the bias in the *I method* resulting from dismissing the patients in HW set, a subset of the hospitalized inpatients is included in the estimation procedure, those who are more likely to have complete information in their pathways in the short term. This approach does not consider the patients admitted to HW, ICU and DIS sets in the last *d* days, where *d* is calculated as the percentile *P* of the probability distribution of *Z*, estimated at time *t* considering all patients in ICU set:

$$d(t)={F^{-1}}_{{\hat{\theta }}_{Z}^{CI}(t)}(P).$$ (8)

The estimation procedure resembles the *I method* where the datasets HW, ICU and DIS are now replaced with $HWd=HW\backslash \{i|t-{t_{HA}}_{i}<d(t)\},\;ICUd=ICU\backslash \{i|t-{t_{HA}}_{i}<d(t)\}$, and $DISd=DIS\backslash \{i|t-{t_{HA}}_{i}<d(t)\}$. The bias is reduced, because the *HWd* set now includes patients with a small probability of being transferred to ICU, at the expense of estimating with fewer observations in *ICUd* and *DISd* sets.

## 3. Simulation studies

Two simulation studies have been carried out to compare the estimation methods presented in Section 2, and to determine their impact on the predictions of bed occupancy obtained using those estimated distributions and probabilities as simulation inputs. In particular, the goal is to test the performance of the proposed estimation methods in Subsection 2.2 (*NP method*) and Subsection 2.3 (*EM method* and *EMNP method*), and their comparison with the methods in Subsection 2.4 (*CI method*, *I method* and *IP method*) that dismiss incomplete information. In this section, we describe the simulation model and the experimental design that have been carried out to assess the accuracy of both the estimation of *p*_*WI*_ and *F*_*Z*_(*z*) and the prediction of hospital resources needed to care for all patients, specifically the number of ICU beds required. The results related to the estimation accuracy are shown in Subsection 4.1 and the impact on the precision of the predictions, in Subsection 4.2. All methods and simulations have been programmed using Python 3.7.

This section is organised as follows. We first present the mathematical modelling of hospital dynamics using a DES model in Subsection 3.1. Subsection 3.2 describes how the DES model simulates the patient arrival process in order to generate different pandemic waves. In Subsection 3.3, we explain how to simulate the pathway and LoS for each patient at the hospital. Moreover, in Subsection 3.4, we present how to generate the remaining pathway and LoS of patients that are admitted in the hospital at specific time *t* and for those who will arrive in the future. The latter allows different scenarios to be projected into the future based on the hospital’s situation at a specific point in time during the pandemic.

### 3.1. The discrete event simulation model

A DES model is developed to assess the accuracy of the estimators. DES models create entities that are transformed by several processes until they exit the modelling system. In our simulation model, the entities are the COVID-19 patients and the processes are the health care received in the hospital ward and/or ICU. In this way, the DES model is able to reproduce the hospital admission of patients during a pandemic wave and the trajectory in the hospital for each patient. The simulation model represents patient flow through the different hospitalization routes; that is, the area enclosed by dashed lines in Fig 2.

The system is described by a set of state variables, which provide at any time a complete representation of the simulated system, and the set of events, which modify the value of the state variables. We consider two global state variables, number of beds occupied by COVID-19 patients in hospital wards and the ICU, and two patient-dependent state variables, the admission place at time t (ward without a previous stay in ICU, ICU and ward after transferral from ICU) and the time at which patient enters the current admission place.

**Fig 4:**
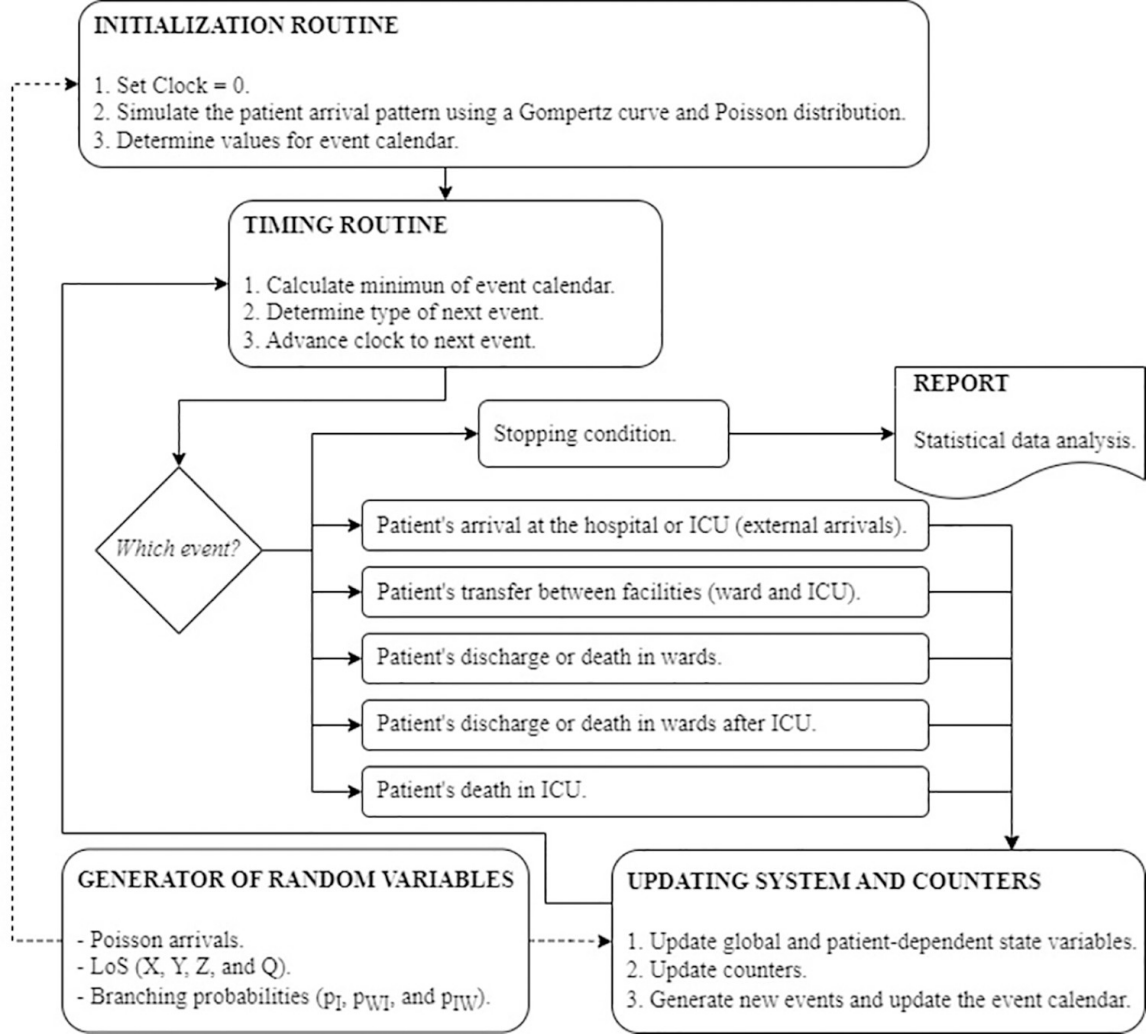
Discrete event simulation model. Flow diagram of the main components of the DES model highlighting the five events that modify the two types of state variables.

The events that modify the state variables are the following five: a new patient admission to the hospital, a patient transfer from ward to ICU, a patient discharge in ward, a patient discharge in ward after ICU and a patient discharge in ICU. Fig 4 outlines the DES model of the health system. A complete description of the DES model, and how each state variable is updated as each type of event occurs is presented in [12].

### 3.2. Patient arrival process

Let *T*_*End*_ be the simulation horizon time for the pandemic wave, and *G*(*t*) the cumulated number of hospitalized patients at time *t* for *t* = 1,…,*T*_*End*_. In this study, *G*(*t*) is simulated using Population Growth (PG) models. This methodology provides methods for modelling the number of cumulative positive cases, hospitalizations, and other pandemic variables. Some examples of PG models that have been found in the literature are the Gompertz [45], the Richards [46], the Stannard [47], and the logistic model [48]. Gompertz model shows a better fit to data of daily COVID-19 new cases as well as better predictive capacity than other PG models [12]. Therefore, the arrival of patients at the hospital are generated using the Gompertz model, via the equation proposed by Zwietering et al. [49] who rewrote the original one [45] to ease the biological interpretation of its parameter. The arrival curve, *G*(*t*), is generated with the following Gompertz model:

G(t)=5000exp(−exp(2.0743−0.0678t)) (9)

**Fig 5:**
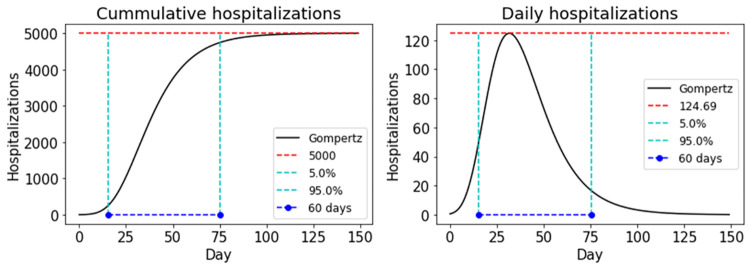
Gompertz curve generated to model a pandemic scenario. This scenario has 5,000 cumulative hospitalizations and 60 days of duration. The left-hand side shows cumulative hospitalizations for the selected scenario while the right-hand side shows daily ones, that is, the derivative curve.

The selected curve, *G*(*t*), in Eq (9) models a cumulative number of 5,000 patients and a duration of 60 days, where duration is defined as the time elapsed from the admission of 5% to 95% of the total number of patients (see Fig 5).

From the Gompertz-type hospitalization curve the expected number of daily hospitalizations is calculated as λ(*t*) = *G*(*t*)−*G*(*t*−1). The number of daily hospitalizations at day *t*, *H*(*t*), for *t* = 1,…,*T*_*End*_, is simulated from a Poisson distribution with mean λ(*t*):

$$P(H(t)=k)=\frac{e^{-\lambda (t)}{\lambda (t)}^{k}}{k!},t=1,\ldots ,T_{End}$$ (10)

Therefore, in each of the simulated scenarios, patient arrival pattern is different.

### 3.3. Flow of patients in the hospital

For each patient arriving at the hospital, a pathway is simulated reproducing the patient pathway outlined in Fig 1. Each patient can be admitted to the hospital ward or directly to the ICU. The probability of direct admission to ICU upon arrival is *p*_*I*_ = 0.028. Besides, those patients admitted to the wards may worsen their health status and require the transfer to the ICU. The probability of a patient initially admitted to a ward requiring transfer to ICU was set *p*_*WI*_ = 0.088. From both hospital ward and ICU, patients can die, so they abandon the system, or they can be discharged after improving their health status. In the last situation, patients in the ICU would be transferred to the hospital ward until they get over the disease. The probability of a patient being transferred from ICU to hospital ward is *p*_*IW*_ = 0.816.

In the simulation experiments, probability distributions for the LoS are assumed to be Weibull *W*(*α*, *β*), where *α* is the scale parameter and *β* is the shape parameter, and time is measured in days: LoS in the hospital ward of a patient not needing ICU, variable *X*, is distributed as *W*(10.2, 1.25), the time spent by a patient in the hospital ward before transfer to the ICU, variable *Z*, is distributed as *W*(4.1, 1.15). In addition, the LoS of a patient in the ICU, both variables *Y* and *D*, are distributed as *W*(17.3, 1.1). Finally, the LoS of a patient in hospital ward after being discharged from ICU, variable *Q*, is distributed as *W*(11.85, 1.4).

All these selected values are estimations based on real patients during a COVID-19 pandemic wave [12].

### 3.4. Simulating future hospital patient-flow

At a specific day of a pandemic wave, prediction of the resources needed for patients care, such as ICU beds, might be of interest. In this study, the simulated pandemic wave is referred to as Reference Scenario (RS), and the specific day is called Simulation Starting Point (SSP).

For prediction of bed occupancy at time *t*, the future pathways for the inpatients must be simulated by estimating all the distributions and probabilities involved in the patient’s pathway (see Fig 2) with the information available up to that specific day *t*.

Accurate prediction of ICU bed occupancy relies on the efficient estimates of all the probabilities and distributions in patients flow (see Fig 2). The goal of this study is limited to assess the influence of the estimation of *p*_*WI*_ and the distribution of variable *Z* in predicting ICU bed occupancy. For this reason, and in order to avoid extra variability into the simulation study so the differences in the estimations of bed occupancy and prediction capability are only assigned to the differences in the estimation of *p*_*WI*_ and the distribution of variable *Z*, patients pathways are simulated using the estimated values of *p*_*WI*_ and the distribution of variable *Z* using the methods of Section 2, the other probabilities and distributions involved in patients pathways (see Fig 2) are considered as known, and given by the models in Subsection 3.3.

Future pathways must be simulated for the three possible types of patients: patients in hospital ward at SSP day, patients currently admitted to the ICU at SSP day, and future patients admitted in the coming days.

A hospital pathway is simulated for each patient *i* currently in hospital ward for *t*_*i*_ days as follows. The patient is admitted to the ICU with probability

$${\hat{p}}_{WI,i}(t)=\frac{\left(1-{\hat{F}}_{Z,t}(t_{i})){\hat{p}}_{WI}(t\right)}{\left(1-{\hat{F}}_{Z,t}(t_{i})){\hat{p}}_{WI}(t)+(1-F_{X}(t_{i}))(1-{\hat{p}}_{WI}(t)\right)},$$

where ${\hat{p}}_{WI}(t)$ and the function ${\hat{F}}_{Z,t}(z)$ are the estimations computed with the methods in Section 2. If the patient requires ICU admission, then the simulated time in hospital ward left to ICU admission is *z*_*i*_−*t*_*i*_, where *z*_*i*_ is generated from the conditional distribution *Z*|*Z*>*t*_*i*_, that is, ${(\hat{F}}_{Z,t}(z_{i})-{\hat{F}}_{Z,t}(t_{i}))/\left(1-{\hat{F}}_{Z,t}(t_{i})\right)$. If the patient *i* does not require ICU care, the hospital discharge will occur after a time *x*_*i*_−*t*_*i*_, where *x*_*i*_ is sampled from the conditional distribution *X*|*X*>*t*_*i*_, that is, (*F*_*X*_(*x*_*i*_)−*F*_*X*_(*t*_*i*_))/(1−*F*_*X*_(*t*_*i*_)).

The pathway of patients in ICU at SSP day is generated as in Subsection 3.3. For the simulation of future inpatients, the arrival curve *G*(*t*) must be previously estimated. In this study the patient arrival process is the same as the one used for the simulated pandemic wave RS and given by Eqs (9) and (10), to avoid introducing more variability into the simulation study. Once the future patient arrives, the pathway is simulated as in Subsection 3.3 using the models therein, except *p*_*WI*_ and the probability distribution of variable *Z* which are estimated with the methods in Section 2.

This simulation can be performed using different days as SSP. Subsection 4.2 shows the results obtained with four different days. It can be observed how the predictions change as more data is available for the estimations.

## 4. Results

This section presents the results obtained in the two simulation studies. First, in Subsection 4.1, we show the accuracy of the estimators as the pandemic progresses. Second, in Subsection 4.2, we include the impact of the estimates of *p*_*WI*_ and the distribution of variable *Z* on the simulation output. Specifically, we study the accuracy obtained in predicting the number of occupied ICU beds during a generated pandemic scenario.

In the use of the *IP method*, a value for the P percentile is needed in the computation of *d* in Eq (8). The two following percentiles have been chosen for the estimations: 50^th^ percentile (*IQ2 method*) and 75^th^ percentile (*IQ3 method*). In the use of the *EM* based procedures in Subsection 2.3 *(EM method* and *EMNP method)*, we set $\varepsilon_{X}=\varepsilon_{Z}=\varepsilon_{p_{WI}}=0.01$ as stop criteria of the EM algorithm.

### 4.1. Estimation accuracy

To assess the accuracy of the estimations we generated 100 different pandemic waves, with *T*_*End*_ = 150, according to Subsection 3.2 and Subsection 3.3. In each scenario and for each day *t = 1*,*…*,*80*, we estimated the probability of admission to ICU from hospital ward *p*_*WI*_, and the distribution of variable *Z*, time to transfer to ICU from wards, with the information provided by the corresponding *n(t)* patients during the first 80 days. In order to compare the methods to estimate *F*_*Z*_(*z*), we computed $\hat{\mu_{Z}}(t)$ the mean of the estimated distributions ${\hat{F}}_{Z,t}(z)$ at time *t* and compared it to the real mean, *μ*_*Z*_ = *E*(*Z*) = 3.9 days. Besides, we approximated the integrated squared error (ISE) between the estimated curve ${\hat{F}}_{Z,t}(z)$ and the true distribution function *F*_*Z*_(*z*).

**Fig 6:**
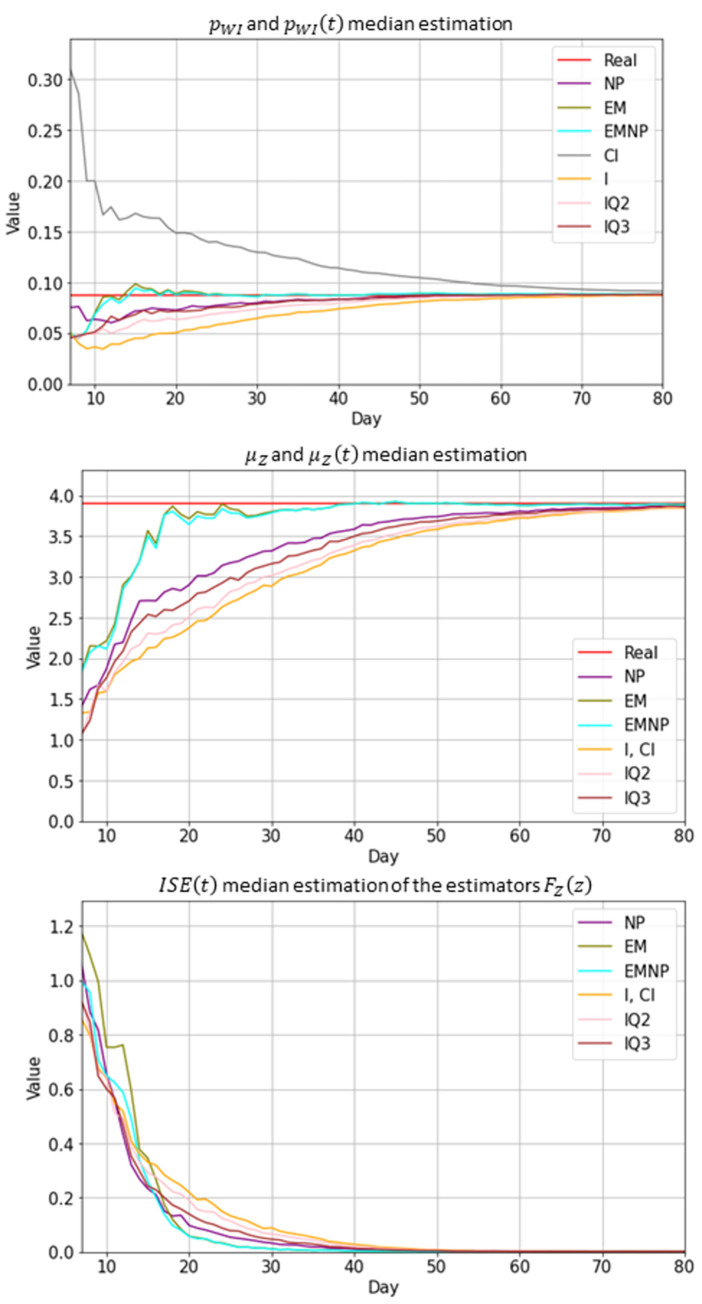
The evolution over time of the estimations using different methods. Median of the estimations of the probability *p*_*WI*_ (top), median of the estimated mean times in hospital ward until admission to ICU, *μ*_*Z*_ (center), and ISE of the estimators of the distribution function of variable *Z* (bottom) over time t with all the methods, computed with 100 simulated pandemics. The real values of *p*_*WI*_ and *μ*_*Z*_ are included for reference (red line). The horizontal axes represent the time (days) during the pandemic.

**Fig 7:**
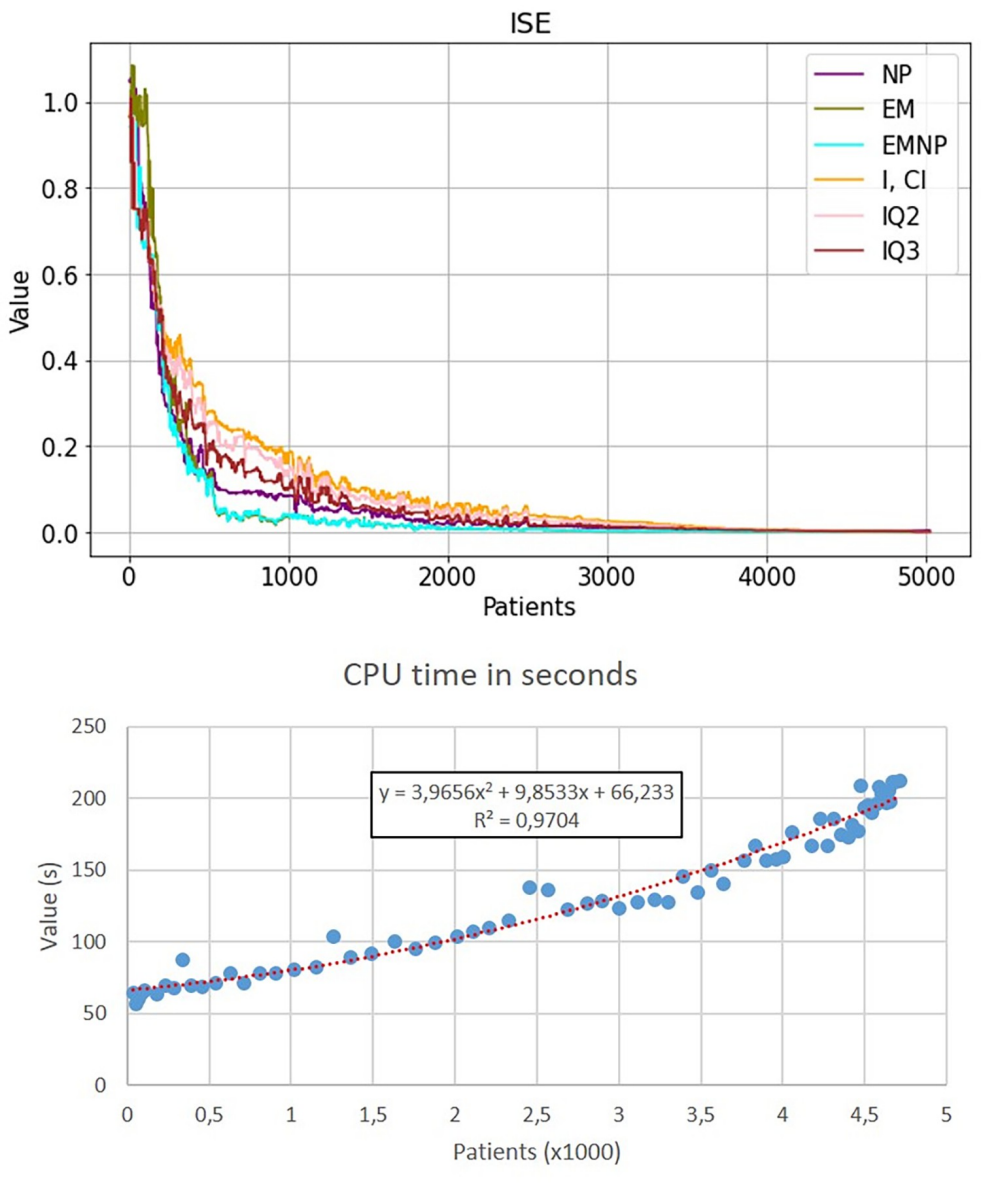
Illustration of convergence in experiments. Relationship between the error and the sample size with which the parameter estimation is done (top), and CPU time (in seconds) needed to obtain parameter estimations using the EMNP method as a function of the sample size (bottom).

Fig 6 shows the evolution over time of the estimation of *p*_*WI*_, the estimated mean value of variable *Z* and the ISE of the estimators of *F*_*Z*_(*z*). The results show the median value of the 100 scenarios for each day. All methods provide results that converge to the true value of the estimated parameter (red color). Note that the sample size *n(t)* increases with *t*, so this convergence shows the consistency of the methods. The top graph in Fig 7 shows the convergence of the ISE as the sample size increases. The bottom graph in Fig 7 displays the computational effort to calculate the EMNP estimator, which moderately increases with the sample size. The computer used in the experiment was an Intel (R) Xeon (R) CPU E5-1630 v4 3.70GHz with 64.0 GB RAM. Even for large sample sizes, such as 5000, it takes about three minutes to get the estimation, which is affordable because it only needs to be done once a day, in order to predict the bed occupancy level.

Both the *EMNP method* and the *EM method* have a fast convergence in all simulated cases, which turns out to be relevant when the simulation model is used as a prediction tool for the resources needed in the future, as we expose in the next subsection. The *NP method* provides the third best results, improving alternative naïve methods. Nonetheless, the bias of these latter estimators, due to information from patients in hospital ward is dismissed, tends to fade away at advanced stages of the pandemic, as the proportion of these censored observations decreases. It should also be highlighted that the estimator *IQ3* outperforms the estimator *IQ2*.

### 4.2. Impact on the simulation output. Bed occupancy prediction accuracy

Simulation is used to predict the future bed occupancy level during the course of a fixed generated pandemic wave (RS), when the pandemic is on the 15^th^, 20^th^, 25^th^, and 30^th^ days (SSP). For each SSP, we generated 500 future courses of the pandemic by simulating future hospital patient-flows, as described in Subsection 3.4. The predictions of ICU beds demand are obtained by the statistical analysis of the output of these 500 runs. The corresponding bed occupancy forecasts for each method are compared with those obtained from 500 future developments of the pandemic generated by simulating patient pathways based on the true values of the parameters and probabilities in Subsection 3.3.

**Fig 8:**
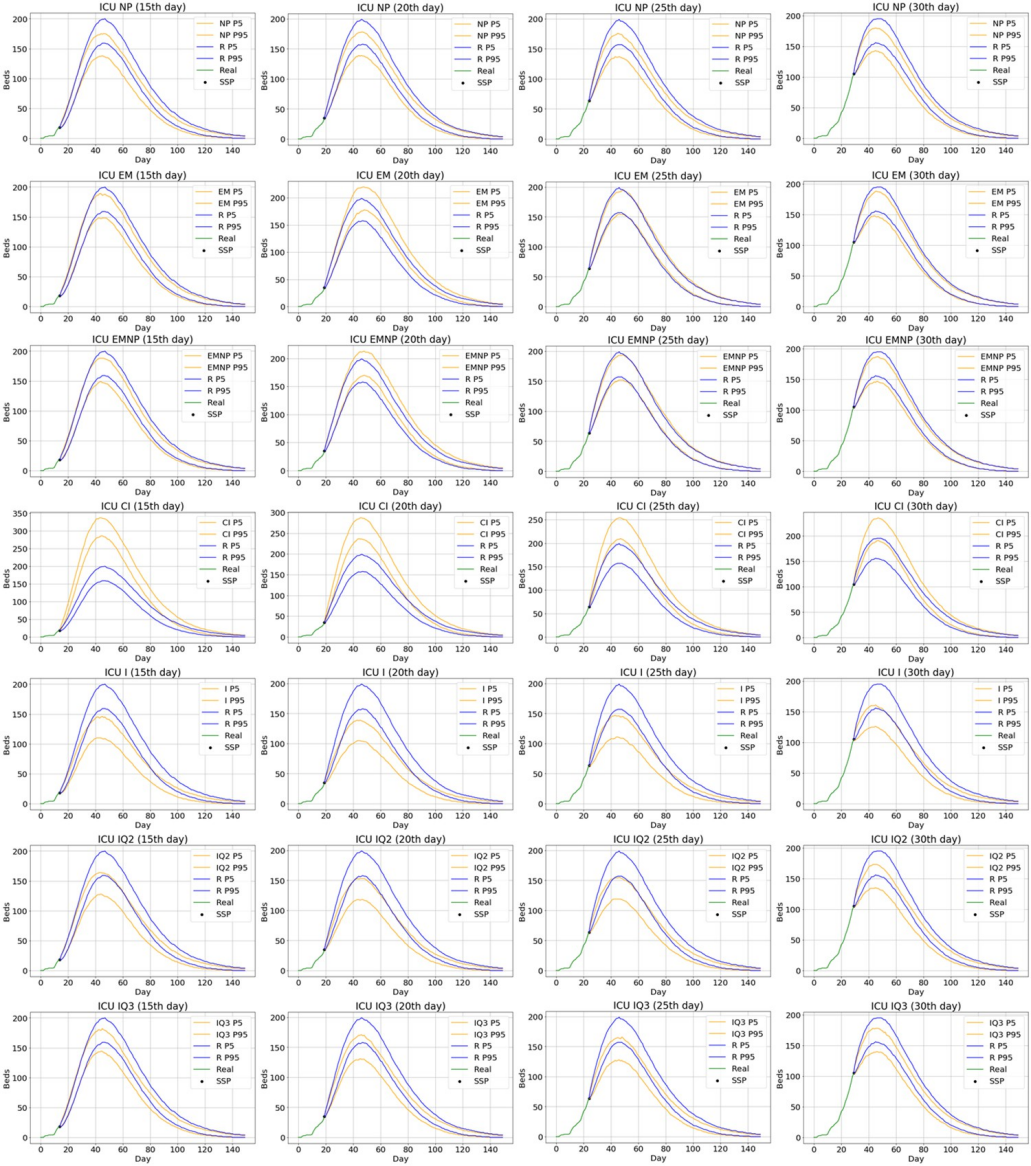
Twenty-eight predictions of ICU bed occupancy considering all methods from 4 different days. Prediction (5^th^ and 95^th^ percentiles) of ICU bed occupancy on the 15^th^, 20^th^, 25^th^, and 30^th^ days of the pandemic with all the methods compared to prediction (5^th^ and 95^th^ percentiles) with actual parameters, denoted with letter R.

Fig 8 shows twenty-eight predictions of ICU bed occupancy considering all methods from 4 different days (15^th^, 20^th^, 25^th^, and 30^th^). Note that these days are quite far away from the peak occupancy (45^th^ day), with expected ICU bed occupancy of 176 beds and 90% centred prediction interval (154,198). The green line in each graph represents the evolution of the simulated pandemic up to the SSP (black dot). For each method, the 5^th^ percentile (P5) and the 95^th^ percentile (P95) of the predicted ICU occupancy levels are plotted using orange lines, whereas the blue lines represent the 5^th^ and 95^th^ percentiles of the predictions when pandemic is simulated using the real parameter values (denoted by the letter R). As the pandemic progresses, the predictions of ICU bed occupancy of all methods approach the real occupancy rate. However, the *EMNP method* is the closest one for all the four different estimating days. Based on the results, we can also conclude that the *EM method* performs almost as well as the *EMNP method*. Besides, the *NP method* again improves the naïve methods that do not use all available information, and the *IQ3 method* has a better behavior than the *IQ2 method*. Finally, the *IC method* clearly overestimates ICU bed occupancy while *I method* underestimates ICU bed occupancy for all the SSP times considered.

**Fig 9:**
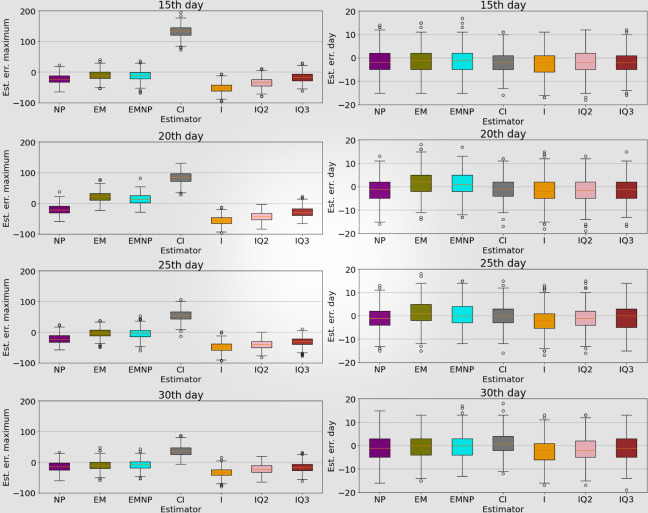
Estimation errors between the predictions when simulating with each estimation method and with actual values. Analysis of the maximum bed occupancy in the ICU, and the day on which the maximum occurs. For days 15^th^, 20^th^, 25^th^, and 30^th^, the estimation errors are shown for each of the 500 simulations between the values obtained with each method and the predictions with the real parameters.

In addition, we have also studied the errors in the predictions of the maximum number of occupied ICU beds and the day on which the maximum occurs. Fig 9 shows boxplots representing the estimation errors between the predictions when simulating with each estimation method and the predictions when simulating using the actual values of the parameters, calculated for each of the 500 simulations at different times (15^th^, 20^th^, 25^th^, and 30^th^ day). Positive differences indicate an overestimation while negative differences indicate an underestimation. When predicting the maximum number of occupied ICU beds, we can observe that the predictions improve as the prediction day advances, and the *EM* method and the *EMNP* method outperform all other approaches, with average errors of 10.184 and 11.574 beds respectively when estimating the 15^th^ day, and 9.204 and 8.628 beds when estimating the 30^th^ day. However, the results for the estimation of the day of maximum occupancy are very similar for all methods and all the days considered.

## 5. Discussion

In this work, we consider a DES model to forecast ICU bed demand via simulation of inpatient’s future pathways. The simulation of patients’ flow is carried out when all the distributions and branching probabilities in the patients’ pathways are estimated. We introduced different methods to estimate efficiently these probabilities and lengths of stay, and showed how to apply them to estimate the probability that an inpatient in hospital ward will be admitted to ICU, and the distribution of the time in hospital ward until admission to ICU. The proposed methods can also be applied to estimate all other distributions and probabilities that define the pathway of a patient, such as the probability of dying in ICU or the time in ICU until discharge. The great advantage of the proposed methods is that the estimation does not rely on published data across heterogeneous populations or health system’s historical data, but on the real patients admitted to the hospital during the period when prediction is of interest. If information is updated frequently, then hospitalization and bed demand forecasts will be more accurate. The second advantage is that partial information provided by inpatients still in hospital at the time of estimation is included in the estimation procedures, which increases efficiency and reliability of the results. Note that the main challenge of using up-to-date patient-level information is that data provided by patients still in hospital ward is censored, as the future path of these patients remains unknown. Methods that take advantage of the partial information associated to these patients using mixture cure models (MCM) have been shown to be more efficient that naïve methods that do not use survival analysis techniques.

The EM and EMNP estimators can be applied in other contexts, for example, to estimate the parameters of stochastic compartmental models used to represent the spread of a pandemic [16, 18, 50]. Some compartmental models extend the original SIR model introducing more compartments such as Exposed, Quarantined, Hospitalized, etc. [50, 51]. The patient pathway through these compartments can be similar to those represented in Fig 3, and then susceptible to applying the estimators presented in this research. For example, an Infected patient can transit to the Recovery compartment or to the Hospital compartment [18]. Our estimators can estimate the probability distributions of both time until recovery and time to hospital admission, and transition probabilities, by using up-to-date data, which allows for model calibration at the first stages of the pandemic when the uncertainty is the greatest. Other context of possible application of both estimators is reliability, where data coming from both laboratory tests and field observation are usually censored. Maximum-likelihood-based estimators have been proposed in the literature to deal with these data, see for example [52, 53], which assume a type-II censored scheme, which differs from ours.

The simulation results for the different scenarios show that the *NP method*, that does not assume any parametric form for the distribution of the times, not only provides more flexibility to the model but it converges faster than the parametric approaches. Note that fast convergence is relevant to have reliable estimates at early stages of the pandemic, so the simulation model could be used as a prediction tool for the hospital bed occupancy in the near future. The other methodology that gives very good results is the proposed *EMNP method*, which combines the nonparametric approaches based on the MCM of the aforementioned *NP method*, and assumes a parametric form for the distribution of the lengths of stay, with parameters estimated using the EM algorithm. The behavior of this new *EMNP method* has been assessed in a simulation study. As expected, the *EMNP method* outperforms the other approaches, as the distribution of the simulated data fulfils the parametric assumption in the *EMNP method*.

It is important to note that a variety of limitations exists for the proposed methods. One limitation concerns the simulated pathway for the patients, specifically, the number of admissions to ICU. In the simulated model, a patient is assumed to require ICU once at most. Although this is the most common case for inpatients in a hospital, it might not be realistic for all the subjects. The model can be extended to include more than one admission to ICU. However, increasing the possible number of times in ICU would make the model to become considerably complex.

A second limitation is that the proposed methods do not consider level-patient characteristics like infection severity, age, comorbidity status and diagnostic testing results. All the methods can be extended to incorporate these characteristics in the estimations as covariates. If only one covariate is to be included, the *NP method* and the *EMNP method* can be extended following Safari et al. [30, 33]. When there are many covariates, the sparseness of data gives rise to the well known “curse of dimensionality”, which implies that massive amounts of data will be required for accurate estimate as the number of covariates increases. Different approaches are available in the literature, which enable handling multiple covariates when estimating nonparametrically under censoring [54–56]. Alternative approaches to extend the *NP method* and the *EMNP method* to multiple covariates are the proportional hazards model [35–39] or the accelerated failure time model [40–44]. However, these extensions are beyond the scope of this study and considering these approaches is left for future work.

The efficiency of the proposed methods depends strongly on the quality of the patient-level information provided by the hospital electronic health record systems. The information should be uploaded frequently into the system, and the model updated accordingly. Notwithstanding, the proposed methods are more dynamic and adaptive than any other approach based on historical data. They are flexible and can be updated when new data are available.

In conclusion, the proposed *NP method* and *EMNP method* provide a useful, efficient, adaptive and easily applicable methodology for estimating the distribution times and branching probabilities in inpatients’ pathways. These methods achieve good performance without relying on comparable historical data that may not be available or may not be realistic. In addition, they are flexible and can be further extended to accommodate multiple patient-level characteristics. The provided estimates can be used subsequently in DES for modelling the demand for critical care beds. As a result, the proposed methods are useful tools to forecast bed occupancy, and we hope they are helpful to improve making decisions in hospital management.
